# A genome-wide association study of arabinoxylan content in flour of triticale (×*Triticosecale* Wittmack)

**DOI:** 10.3389/fpls.2026.1809699

**Published:** 2026-04-27

**Authors:** Maria Chiara Piro, Hans Peter Maurer, Hilde Muylle, Steven Maenhout, Geert Haesaert

**Affiliations:** 1 Department of Plants and Crops, Faculty of Bioscience Engineering, Ghent University, Ghent, Belgium; 2 University of Hohenheim, State Plant Breeding Institute, Stuttgart, Germany; 3 Plant Sciences Unit, Flanders Research Institute for Agriculture, Fisheries and Food (ILVO), Melle, Belgium

**Keywords:** arabinoxylan, dietary fibre, end-use quality, GWAS, triticale

## Abstract

The adoption of triticale (×*Triticosecale* Wittmack) by the food market is still limited due to its inferior technological quality compared to wheat when it comes to bread-making. However, there is continued interest in the use of triticale flour for food purposes owing to its rich content of bioactive compounds, particularly dietary fibre (DF). The primary constituent of triticale DF is arabinoxylan (AX). The intake of AX is associated with several health benefits, from prebiotic function to the regulation of post-prandial metabolism. Genetic research to identify breeding targets to improve the quality of triticale flour remains scarce. Particularly so in relation to bioactive compounds such as DF. In the present study, a diverse triticale collection was genotyped by means of DArTseq technology and phenotypically characterised in terms of total AX (TOT-AX), water-extractable AX (WE-AX), water-unextractable AX (WU-AX) contents in flour, and the proportion of WE-AX to TOT-AX (WE/TOT-AX). Seven significant marker-trait associations (MTAs) were found. Two MTAs located on the short arm of chromosome 6B are consistent with a known QTL for WE-AX of bread wheat (*Triticum aestivum*). Three MTAs, one located on chromosomes 4R, and two located on chromosome 5R are consistent with research on wheat-rye translocation and addition lines.

## 1 Introduction

Triticale (×*Triticosecale* Wittmack) is an amphiploid cereal crop which originated from the man-made cross of either hexaploid wheat (*Triticum aestivum*, 2n = 42, BBAADD) or tetraploid wheat (*T. durum*, *T. dicoccoides*, 2n = 28, BBAA) with rye (*Secale cereale*, 2n = 14; RR; Oettler, 2005). The first chromosome doubled progeny of such wheat × rye crosses is termed “primary” triticale, whereas the progeny of subsequent crosses between a primary triticale and wheat, rye, or another triticale is termed “secondary” triticale (Lukaszewski and Gustafson, 1987; Oettler, 2005). Triticales of different ploidy levels have been developed in the course of triticale breeding, including tetraploid triticale (2n = 28, AARR, BBRR), hexaploid (2n = 42, BBAARR) or octoploid (2n = 56, BBAADDRR; Feldman and Levy, 2023; Łapiński, 2002; Lukaszewski and Gustafson, 1987; Oettler, 2005). Eventually, secondary, hexaploid triticale was the form that caught on as a profitable agronomic species, owing to its higher fertility and meiotic stability (Feldman and Levy, 2023; Oettler, 2005). Among the available triticale germplasm, it is possible to distinguish between “complete” or “substitute” triticale. This distinction is made based on whether the A, B, and R subgenomes comprise a full set of chromosomes or are partially substituted by chromosomes of any of the other subgenomes (Ammar et al., 2004; Feng et al., 2019; Sodkiewicz et al., 2011). Studies describing “substitute” triticale often refer to breeding material developed at CIMMYT, Mexico (International Maize and Wheat Improvement Center), whereas triticale grown in Europe is thought to be “complete” triticale (Ammar et al., 2004; Chen et al., 2019; Feng et al., 2019; Tams et al., 2004).

Although the original intention behind the development of triticale was to combine the technological properties of wheat with the hardiness of rye, the adoption of triticale by the food market remains hampered by the inferior technological quality of the triticale grain and flour with respect to bread-making (Camerlengo and Kiszonas, 2023; Mergoum et al., 2019; Oettler, 2005; Woś and Brzeziński, 2015). In fact, the triticale grain is generally characterised by several traits that make it less suitable for milling and bread-making such as a very soft grain texture, low wet gluten content, low glutenin content, and low falling number (Aprodu and Banu, 2017; Camerlengo and Kiszonas, 2023; Chavoushi et al., 2022; Kaszuba et al., 2024; Navarro-Contreras et al., 2014; Woś and Brzeziński, 2015). Nevertheless, there is a continued interest in using triticale flour for food applications due to the higher biological value of its protein compared to wheat protein, as well as its higher levels of micronutrients and bioactive compounds, particularly insoluble and soluble dietary fibre (DF; Aprodu and Banu, 2017; Camerlengo and Kiszonas, 2023; Fraś et al., 2016; Heger and Eggum, 1991).

DF refers to the total of resistant starch, non-starch polysaccharides such as cellulose, hemicelluloses (including xylans, mannans, mixed-linkage glucans, and xyloglucan), pectin, fructo-oligosaccharides (i.e., fructans), and Klason lignin (Stephen et al., 2017; Theander et al., 1994). All these carbohydrate polymers and Klason lignin share the characteristics that they are neither digested nor absorbed in the small intestine and that they provide a beneficial physiological effect (EFSA Panel on Dietetic Products, Nutrition, and Allergies (NDA), 2010; Stephen et al., 2017). In cereals, hemicelluloses such as arabinoxylan (AX) and (1 → 3)(1 → 4)-β-D-glucan are the major DF constituents, depending on the species considered (Rakha et al., 2011; Ward et al., 2008). Similarly to its wheat and rye ancestors, the major DF component of triticale is AX, which accounts for about 40% of the total DF content (Rakha et al., 2011; Ward et al., 2008).

AX constitutes a heterogenous population of molecules characterised by a backbone of (1→4)-β-D-xylopyranosyl (Xyl*p*) residues which can feature mono-substitutions with α-L-arabinofuranosyl (Ara*f*) residues on the *O*-2, and *O*-3 positions, or di-substitutions on the *O*-2 and *O*-3 positions simultaneously (Saulnier et al., 2007). The Ara*f* residues, in turn, can also be decorated by hydroxycinnamic acids, mainly *trans-*ferulic acid (FA; Li et al., 2008; Saulnier et al., 2007). The degree of polymerisation of the Xyl*p* backbone, the Ara*f* to Xyl*p* ratio, and the degree of substitution by FA vary greatly within the grain depending on grain tissue, as well as between cereal species (Buksa et al., 2016; Chavoushi et al., 2022; Gebruers et al., 2008; Nyström et al., 2008; Rakha et al., 2011; Saulnier et al., 2007).

Triticale AX content and structure in terms of degree of Ara*f* substitution and molecular weight distribution remain poorly characterised, but they are reported to be intermediate to that of wheat and rye (Chavoushi et al., 2022; Rakha et al., 2011). Considering AX content, triticale resembles its wheat ancestor more closely than the rye counterpart. Rakha et al. (2011) reported a range of triticale wholemeal total AX (TOT-AX) content between 5.9 to 7.5% of dry matter (% dm), depending on variety and trial location. The reference wheat variety had a TOT-AX content in the range of 6.0-6.4% dm depending on trial location, whereas the rye reference variety had a TOT-AX content in the range of 8.4-8.8% dm (Rakha et al., 2011). These authors focussed on TOT-AX content of wholemeal, whereas Chavoushi et al. (2022), provided a description of the content in triticale flour of water-soluble pentosans – which are comprised by almost entirely water-extractable AX (WE-AX). In their study, the content of water-soluble pentosans ranges between 0.71 and 1.25% dm, whereas the content of water-soluble pentosans of the wheat and rye reference varieties used in the same study were 0.94 and 2.22% dm respectively.

Genome-wide association studies (GWAS) are widely used to identify molecular breeding targets (Tibbs Cortes et al., 2021). In the case of triticale, association studies are not as common as for other crop species, possibly because of its peculiar genomic structure and the lack of an appropriate reference genome. Nevertheless, DArTseq genotyping combined with a follow-up GWAS has already uncovered several marker-trait associations (MTAs) that relate to agronomic and quality traits (De Zutter et al., 2024; Galiano-Carneiro et al., 2018; Losert et al., 2017a; Neuweiler et al., 2020, Neuweiler et al., 2021; Niedziela et al., 2012; Trini et al., 2021; Zustovi et al., 2025). DArTseq is a reference-free genotyping method, meaning that SNP calls are not made by comparing the amplified sequences against a reference genome (Jaccoud et al., 2001; Kilian et al., 2012). Thus, it is a versatile method that has been successfully applied to a variety of species, regardless of the availability of a reference genome (Ali Koura et al., 2024; Aznar-Fernández et al., 2020; Barilli et al., 2018; Boczkowska et al., 2020; Correa Abondano et al., 2024; Perez et al., 2021).

AX is already considered as an interesting breeding target for wheat (Török et al., 2019; Ward et al., 2008). The interest lies in its prebiotic properties (i.e., fermentability by the intestinal microflora) and the associated health benefits such as the positive regulation of postprandial glucose and insulin levels, and the antioxidant function thanks to the FA sidechains (Broekaert et al., 2011; Garcia et al., 2007; Lu et al., 2000, Lu et al., 2004; Pereira et al., 2021). In the context of AX-targeted breeding, it is interesting to identify breeding targets for TOT-AX content as well as its soluble and insoluble fractions. In fact, WE-AX and insoluble AX can reach different parts of the colon where they select for specific bacterial species (Broekaert et al., 2011; Paesani et al., 2020; Pereira et al., 2021; Rumpagaporn et al., 2015). However, the ranges of TOT-AX or WE-AX contents in the available triticale germplasm are still poorly characterized. Rakha et al. (2011) and Chavoushi et al. (2022) investigated only eight and twelve triticale varieties, respectively. Furthermore, genetic targets to breed for higher content of bioactive compounds such as DF in triticale have not yet been described. Therefore, the present study aims to characterise the ranges of TOT-AX or WE-AX contents in a diverse triticale collection, and to uncover MTAs related to the AX content in flour of triticale, thus offering breeders practical tools for improving AX levels via selection.

## 2 Materials and methods

### 2.1 Plant material and field trial conditions

A collection of 118 (×*Triticosecale* Wittmack) winter triticale accessions as described in detail by De Zutter et al. (2023) was used in this study. Briefly, the collection was assembled with the aim of representing the global winter triticale germplasm and thus included accessions from North America as well as from Europe. The collection comprised 101 cultivated varieties and 17 breeding lines (**Supplementary Table 1**).

Two field trials, hereafter referred to as S19/H20 and S21/H22, were conducted at the HOGENT/UGent Bottelare Experimental Farm (latitude 50°57’43”, longitude 3°45’37’’) in the growing seasons of 2019–2020 and 2021–2022 respectively. Both field trials were laid out as a resolvable row-column design with three replications. The triticale collection was sown in micro-plots of 1 m² at a fixed sowing density of 350 kernels/m². Field management followed standard local agronomic practices and mineral fertilisation levels were adjusted based on the results of the soil analysis performed by the Soil Service of Belgium (Heverlee, Belgium). Weather data were retrieved from the local weather station, and included parameters such as daily minimum temperature (°C), daily maximum temperature (°C), and daily total precipitation (mm).

### 2.2 Phenotypic characterization of the triticale endosperm

Endosperm-enriched flour was obtained by milling 100 g of triticale kernels with a Sedimat laboratory mill (Brabender^®^, Duisburg, Germany), which is equipped with a sifter lined with a 150 µm mesh. The kernels were conditioned to 13.5% moisture prior to milling, in order to limit bran contamination of the endosperm-enriched flour.

The TOT-AX and WE-AX contents in the endosperm-enriched flour were determined by a colorimetric assay as described by Kiszonas et al. (2012), with modifications. Namely, each sample set consisted of 15 different triticale flour samples in single technical replicate and one reference flour sample of the wheat variety “Evina” in triplicate, for a total of 18 flour samples per sample set. Analysis of TOT-AX and WE-AX contents of each sample set was performed on the same day. The reaction reagent consisted of 836 ml glacial acetic acid (VWR Chemicals, Leuven, Belgium), 17.48 ml 37% HCl (Thermo Scientific Chemicals, Brussels, Belgium), 38.0 ml 10% m/v phloroglucinol (>99.0%; Merck Life Science BV, Hoeilaart, Belgium) in ethanol absolute (VWR Chemicals), 7.6 ml 1.75% m/v glucose (99+%, anhydrous; Thermo Scientific Chemicals) in demineralised water.

For the determination of TOT-AX content, 20 mg of endosperm-enriched flour was weighed directly in a 50 ml tempered glass test tube. Consequently, 6.0 ml of demineralised water was added to the test tubes, which were then vortexed briefly. A total of 30.0 ml of reaction reagent was added to the test tubes, which were consequently vortexed again to thoroughly homogenise the reaction mixture. The sample set was placed in a boiling water bath for 25 minutes to catalyse the reaction between AX and phloroglucinol, and then transferred to an ice bath for four minutes in order to stop the reaction. After cooling, the tubes were inverted and vortexed to even out the temperature and homogenise the content of the reaction mixture. Finally, about 1 ml of the reaction mixture was transferred to ROTILABO^®^ single-use cuvettes (Carl Roth, Karlsruhe, Germany) and the absorbance at a wavelength of 553 nm was read using a SPECTROstar Nano spectrophotometer (BMG Labtech, Ortenberg, Germany).

For the determination of WE-AX content, 75 mg of endosperm-enriched flour was weighed in a 15 ml single-use falcon tube. The tubes were centrifuged at 30,130 × g for 1 minute to collect all flour at the bottom of the tubes. The tubes were gently tapped to loosen up the compacted flour before adding 10.0 ml of demineralised water. The tubes were vigorously shaken manually to thoroughly homogenise their content, and then placed horizontally on a SSL1 orbital shaker (Stuart; Keison products, Chelmsford, United Kingdom) and shaken at 150 rpm at room temperature for 25 minutes in order to obtain a flour aqueous extract. The water-flour mixture was centrifuged at 1,000 × g at room temperature for 10 minutes in order to separate the flour aqueous extract from the flour solids. Then, 2.0 ml of the supernatant was transferred to 50 ml tempered glass tubes, to which 10.0 ml of the reaction reagent was added. The reaction and spectrophotometric measurements were carried out as described for TOT-AX.

The TOT-AX and WE-AX content values of the flour samples were determined based on a standard absorbance response curve. D-xylose (Merck Life Science BV) solutions (i.e., 0.0, 0.05, 0.10, 0.15, 0.20, 0.25, and 0.30 mg/mL) were used. Duplicate 2.0 ml aliquots of each solution were transferred to a tempered glass tube to which 10.0 ml of the reaction reagent was added. The reaction conditions and spectrophotometric analysis were carried out in the same way as previously described. The TOT-AX and WE-AX contents determined for the reference wheat variety “Evina” were used to calculate a correction factor to account for between-days variation (Equation 1):

(1) $$CF_{j}={\bar{AX}}_{.}/{\bar{AX}}_{.j}$$

Where $CF_{j}$ is the correction factor for day *j*, ${\bar{AX}}_{.}$ is the average TOT-AX or WE-AX content of all the reference flour samples measured during the experiment, and ${\bar{AX}}_{.j}$ is the average TOT-AX or WE-AX content of the reference flour samples on day *j*. The TOT-AX and WE-AX contents of the triticale flour samples were multiplied by the relevant $CF_{j}$ and expressed as % of dry matter (dm). The proportion of WE-AX to TOT-AX (WE/TOT-AX) was calculated as the ratio of WE-AX over TOT-AX contents, and the water-unextractable AX content (WU-AX) was calculated as the difference of TOT-AX and WE-AX contents.

### 2.3 Statistical analysis and data visualization

All data visualisation and statistical analysis were performed using R (version 4.3.1; R Core Team, 2023). All graphical output was produced with custom functions built using the libraries *ggplot2, ggh4x, gridExtra, lemon*, *scales* (Auguie and Antonov, 2017; Brand, 2024; Edwards et al., 2024; Wickham et al., 2023, Wickham et al., 2024).

**Figure 1:**
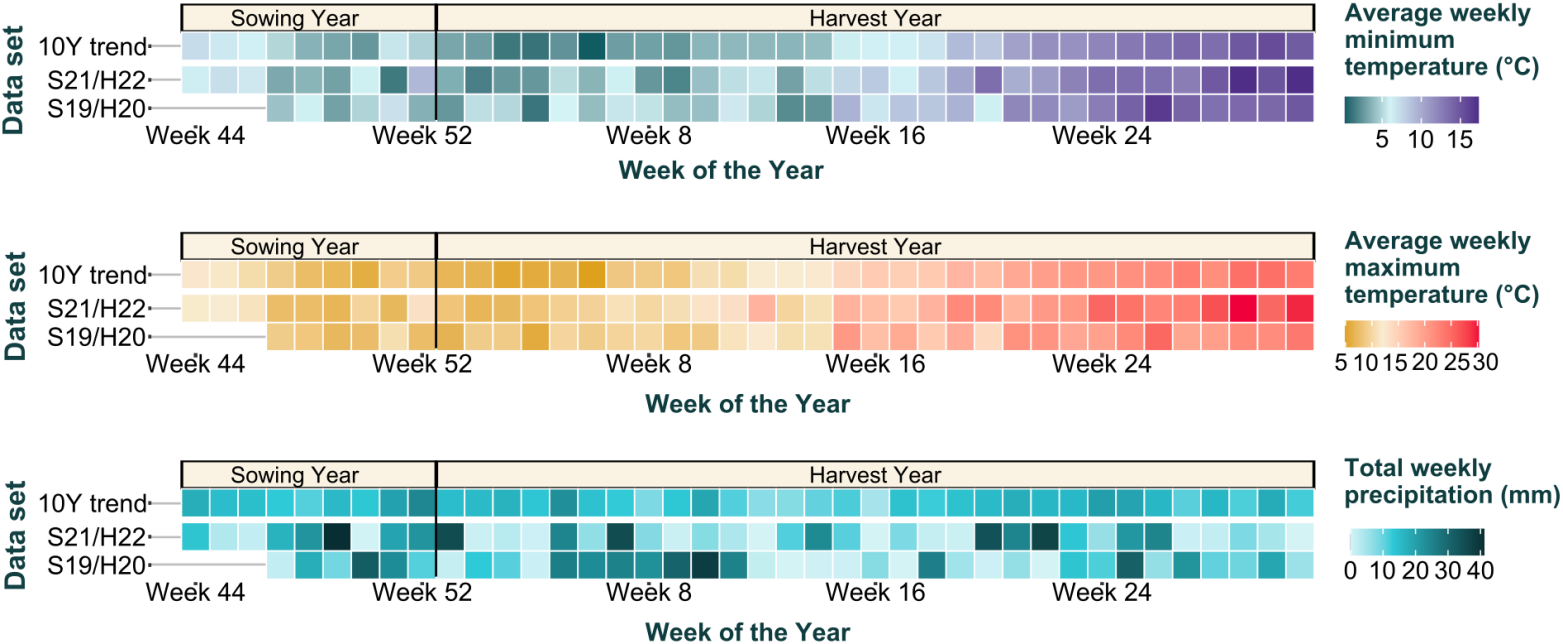
Tile plots of weather parameters including average weekly minimum temperature (°C), average weekly maximum temperature (°C), and total weekly precipitation (mm). Data are aggregated by week and stratified by growing season. Measurements start at the week of sowing and end at the week of harvest. The aggregation in weeks follows the ISO 8601 convention. S19/H20, trial of season 2019-2020; S21/H22, trial of season 2021–2022; 10Y trend, local weather trend over the 10-year period 2012-2022.

The seasonal weather trends were visualised in the style of a tile plot. For this purpose, the collected weather data were aggregated by weeks following the ISO 8601 convention. The aggregated weather parameters used for the visualisation included average weekly minimum temperature, average weekly maximum temperature, and total weekly precipitation. Weather conditions were comparable between the two seasons, nevertheless, in trial S19/H20 the last five trial weeks registered higher total weekly precipitation and lower average maximum temperature in comparison with trial S21/H22 (**Figure 1**).

Summary statistics were calculated for each phenotype and included maximum, minimum, mean, standard deviation, and coefficient of variation (CV). Kendall’s τ rank correlation coefficient between phenotypes was calculated using the *Kendall* library (Kendall, 1938; McLeod, 2022). A mixed-effects linear model fitted with the *lme4* library (Bates et al., 2024) was used to derive genotypic trait values as best linear unbiased estimates (BLUE). The maximal model formula was (Equation 2):

(2) $$Y_{ijklm}=\;\mu +G_{i}+E_{j}+GE_{ij}+EB_{jk}+ER_{jl}+EC_{jm}+\epsilon_{ijklm}$$

Where *Y_ijklm_* is the phenotypic value of the *i^th^* genotype in the *j^th^* environment (i.e., trial), *k^th^* block, *l^th^* row and *m^th^* column, *µ* is the grand mean, *G_i_* and *E_j_*, are the main effects of the *i^th^* genotype and of the *j^th^* environment, respectively, *GE_ij_* is the interaction between *i^th^* genotype and *j^th^* environment, *EB_jk_* is the interaction between the *j^th^* environment and the *k^th^* block, *EC_jl_* is the interaction between the *j^th^* environment and the *l^th^* column, *ER_jk_* is the interaction between the *j^th^* environment and the *m^th^* row, and *ϵ_ijklm_* is the residual. The main effects *G_i_* and *E_j_* were fitted as fixed effects, whereas the interaction terms were fitted as random effects. The optimal model specification for each phenotype was chosen by iteratively removing the random effects until the model with the lowest AIC (Akaike information criterion; Akaike, 1987) value was found.

The conditional and marginal coefficients of determination (R²) relative to each mixed-effects linear model were calculated using the *MuMIn* library (Bartoń, 2023). The conditional R² refers to the variance explained by the model including both fixed and random effects (Equation 3):

(3) $$R_{conditional}^{2}=\frac{\sigma_{f}^{2}+\sigma_{\alpha }^{2}}{\sigma_{f}^{2}+\sigma_{\alpha }^{2}+\sigma_{\epsilon }^{2}}$$

Where *σ²_f_* is the variance of the fixed effects, *σ²_α_* is the variance of the random effects, and *σ²_ϵ_* is the residual variance (Bartoń, 2023). The marginal R² refers to the variance explained by the fixed effects only (Equation 4):

(4) $$R_{marginal}^{2}=\frac{\sigma_{f}^{2}}{\sigma_{f}^{2}+\sigma_{\alpha }^{2}+\sigma_{\epsilon }^{2}}$$

The significance of the main effects *G_i_* and *E_j_* was tested using a type III Wald F-test with Kenward-Roger degrees of freedom (Kenward and Roger, 1997) as implemented in the *car* library (Fox et al., 2024). Broad sense heritability (*H²*) of TOT-AX, WE-AX, WU-AX, and WE/TOT-AX was estimated using the BLUP-based definition by Cullis et al. (2006; Equation 5), as implemented in the library *inti* (Lozano-Isla, 2024):

(5) $$H_{Cullis}^{2}=1-\frac{{\bar{v}}_{\Delta \cdot \cdot }^{BLUP}}{2\sigma_{g}^{2}}$$

### 2.4 Genotyping by DArTseq and SNP panel preparation

The collection was genotyped using the DArTseq platform (Diversity Arrays Technology [DArT], Bruce, Australia; Jaccoud et al., 2001). DNA samples were isolated from leaf material and prepared following the instructions of the genotype provider. The DNA complexity reduction, amplification, sequencing, and SNP calling were carried out by DArT following proprietary protocols.

The SNP data preparation was performed with a custom R script. The SNP panel received from DArT was filtered by call-rate ≥ 0.85 and MAF ≥ 0.05 using the library *dartR* (Gruber et al., 2023). Given that triticale is a mostly-self pollinating species, markers are expected to be mostly in homozygous state for either alleles. Therefore, markers for which either the reference or the alternative allele was present only in heterozygous state were removed, as well as SNP markers with a higher frequency of heterozygous calls than homozygous calls. Imputation of missing data in the SNP panel was performed by k-Nearest Neighbour (kNN) algorithm as implemented in the library *scrime* (Schwender and Fritsch, 2018). The number of neighbours *k* = 5 was specified so as not to include individuals that are genetically too distant from the individual for which imputation is required, and at the same time retain imputation accuracy (Troyanskaya et al., 2001).

Each marker in the original SNP panel as delivered by DArT was assigned a chromosomal coordinate by BLASTn analysis of the DArT marker sequence against the “Chinese Spring” v1.0 (CS) wheat refence genome (The International Wheat Genome Sequencing Consortium [IWGSC] et al., 2018). To adjust the chromosomal coordinates of the DArT marker sequences to the expected genomic composition of triticale, a synthetic reference genome was created by concatenating the FASTA sequences of the A and B subgenomes of the wheat “Renan” reference genome (Aury et al., 2022b) with the FASTA sequences of the rye “Lo7” reference genome (Rabanus-Wallace et al., 2021). The unanchored scaffolds of both the wheat “Renan” reference and the rye “Lo7” reference were retained in the construction of this synthetic reference genome, hereafter referred to as Renan/Lo7. A BLASTn analysis of the DArT marker sequences was performed using the Renan/Lo7 reference and chromosomal coordinates were extracted using custom Python and R scripts. A DArT marker sequence with multiple BLAST hits, was assigned to the chromosomal coordinates of the BLAST hit with maximum length and percent identity. A DArT marker sequence with multiple BLAST hits with equal length and percent identity and on tandem locations on the same chromosome was assigned to the chromosomal coordinates of the first hit. A DArT marker sequence with multiple BLAST hits with equal length and percent identity located on different chromosomes was assigned to the chromosomal location with the highest mean linkage disequilibrium (LD) to its flanking and unambiguously located DArT markers. In the case of such ties, if one of the candidate chromosomal locations was an unanchored scaffold, the DArT marker sequence was marked as “Unassigned”. DArT marker sequences with ties still unresolved past this step were also marked as “Unassigned”.

### 2.5 Genome-wide association study and identification of candidate genes

The GWAS was performed using the Bayesian-information and Linkage-disequilibrium Iteratively Nested Keyway (BLINK) model as implemented in the *GAPIT* library (Huang et al., 2019). Possible deviations in the obtained *p-*values were visually assessed by means of quantile-quantile (Q-Q) plots. Only SNPs with *p-*value < 0.05 after Bonferroni correction for multiple testing were reported as MTAs.

Candidate genes were searched within the range of LD decay around the MTAs output of the GWAS. First, LD was calculated per chromosome as the squared Pearson’s correlation coefficient (*r²*) between pairs of markers using the library *sommer* (Covarrubias-Pazaran, 2025). Then, a locally estimated scatterplot smoothing (LOESS) curve was fit to the calculated LD distributions using the *stats* library (R Core Team, 2023). Lastly, the distance at which intrachromosomal LD decays was calculated as the intersection between the LOESS curve and the threshold value *r²* = 0.2 (Delourme et al., 2013). This physical distance in base pairs was used to define the upstream and downstream boundaries around the MTAs output of the GWAS. LD was not calculated for the pseudomolecule comprising unanchored scaffolds (chrUn), since this pseudomolecule does not represent an actual chromosome. Instead, for chrUn, the average of the calculated LD decay distances was used to define upstream and downstream boundaries.

The genes with functions related to the cell-wall were selected among the candidates using the available gene feature annotations. The gene feature annotation of the rye “Lo7” reference (Rabanus-Wallace et al., 2021) was retrieved from e!DAL (LuxHelmzholtz Z. M, 2020). The gene feature annotations of the “Renan” (Aury et al., 2022b) and “Chinese Spring” v2.1 (Zhu et al., 2021) were retrieved from Genoscope (Aury et al., 2022a) and WHEAT-URGI (The International Wheat Genome Sequencing Consortium (IWGSC), 2024) respectively.

### 2.6 Population structure analysis

The population structure of the triticale collection as initially described by De Zutter et al. (2024), was reassessed by means of a Markov chain Monte Carlo (MCMC)-based non-spatial population structure analysis as implemented in the package *conStruct* (Bradburd, 2024). For this analysis, 195 SNPs were randomly sampled from each chromosome and the group of SNPs lacking a chromosomal projection. Unanchored scaffolds of the rye and wheat subgenomes were pooled together for sampling. The obtained subset comprised about 25% of the filtered SNP panel. The number of layers *k*, loosely equivalent to the number of ancestral populations, was set to four which is consistent with the analysis of De Zutter et al. (2024). One MCMC chain was run for 50,000 iterations. Admixture proportions were used to identify “admixed” genotypes – i.e., genotypes not belonging specifically to any of the population layers. Genotypes with maximum admixture proportion < 0.7 were defined as “admixed”, whereas the remaining genotypes were assigned to a specific layer based on their maximum admixture proportion (Volante et al., 2017).

## 3 Results

### 3.1 Field trials and phenotypic characterization of the triticale endosperm

A wider range of all traits (i.e., TOT-AX, WE-AX, and WU-AX contents and WE/TOT-AX; **Table 1**) was obtained in trial S19/H20 as compared to trial S21/H22. TOT-AX content was negatively correlated with WE/TOT-AX (τ = -0.169, *p-*value < 0.01) and positively correlated with WE-AX (τ = 0.293, *p-*value < 0.01) and WU-AX contents (τ = 0.682, *p-*value < 0.01). WE-AX was positively correlated with WE/TOT-AX (τ = 0.538, *p-*value < 0.01) and uncorrelated with WU-AX content (τ = -0.025, *p-value =* 0.32). Genotype and environment were significant predictors for WE-AX content and WE/TOT-AX (*p-*value < 0.01), whereas only genotype was a significant predictor for TOT-AX and WU-AX content (*p-*value < 0.001). In line with these results, lower estimates of *H²* were obtained for WE-AX and WE/TOT-AX (*H²_WE-AX_ =* 0.68, *H²_WE/TOT-AX_* = 0.60) as compared to TOT-AX and WU-AX (*H²_TOT-AX_* = 0.82, *H²_WU-AX_* = 0.79).

**Table 1:** Summary statistics per phenotype per trial and of the respective distribution of best linear unbiased estimates (BLUE) of genotypic trait values.

Data	Summary statistics	TOT-AX (% dm^a^)	WE-AX (% dm^a^)	WU-AX (% dm^a^)	WE/TOT-AX
S19/H20	Maximum	3.89	1.05	3.04	0.53
	Minimum	1.60	0.44	0.87	0.18
	Mean ± SD	2.19 ± 0.32	0.76 ± 0.11	1.47 ± 0.29	0.33 ± 0.05
	CV	14.66%	15.80%	19.36%	14.57%
S21/H22	Maximum	3.16	0.92	2.43	0.49
	Minimum	1.63	0.41	0.88	0.18
	Mean ± SD	2.12 ± 0.22	0.66 ± 0.10	1.46 ± 0.21	0.31 ± 0.05
	CV	10.35%	14.98%	14.25%	15.28%
BLUE	Maximum	3.29	0.92	2.47	0.40
	Minimum	1.81	0.56	1.08	0.25
	Mean ± SD	2.19 ± 0.21	0.72 ± 0.076	1.47 ± 0.18	0.33 ± 0.031
	CV	9.68%	10.63%	12.42%	9.42%

Summary statistics per phenotype per trial and of the respective distribution of best linear unbiased estimates (BLUE) of genotypic trait values. S19/H20: field trial of season 2019-2020, S21/H22: field trial of season 2021-2022, TOT-AX: total arabinoxylan content, WE-AX: water-extractable arabinoxylan content, WU-AX: water-unextractable arabinoxylan content, WE/TOT-AX: proportion of WE-AX to TOT-AX, SD: standard deviation, CV: coefficient of variation.

### 3.2 Genotyping by DArTseq and SNP panel preparation

The SNP panel obtained by DArTseq comprised 79,121 SNPs, of which a total of 29,633 was retained after filtering by call-rate ≥ 0.85 and MAF ≥ 0.05. When visualising the frequency of heterozygous calls in this subset stratified by subgenome and chromosome group, several loci with high heterozygosity were observed (i.e., maximum > 0.75; **Supplementary Figure 1a**), which is incongruent with a self-pollinating species and the composition of the collection. Therefore, this subset was further filtered based on the expected behaviour of loci in cultivars and advanced breeding lines of a self-pollinating species. Thus, 4,871 markers were removed because either the reference or the alternative allele was present only in heterozygous state, and 6,871 markers were removed because the frequency of heterozygous calls exceeded that of either homozygous classes; yielding a final panel of 17,891 markers (**Supplementary Figure 1b**; **Supplementary Table 3**).

The new BLASTn analysis on the Renan/Lo7 reference increased the completeness of the projections of the DArT marker sequences as compared to the original BLAST analysis on the wheat CS reference (**Supplementary Table 4**). The BLASTn analysis against the Renan/Lo7 reference assigned a chromosomal location to 15,462 out of the 17,891 filtered markers. Of these 15,462 markers, 4,521 were located on the A subgenome, 4,721 were located on the B subgenome, 6,025 were located on the R subgenome, and 195 were located on unanchored scaffolds of the wheat and rye reference genomes. A total of 2,429 markers were marked as “Unassigned”. Of these, 438 markers had multiple perfect or imperfect matches on the Renan/Lo7 reference that could not be unambiguously assigned to a chromosomal location. The remaining 1,991 “Unassigned” markers had a true missing BLASTn match (**Supplementary Table 3**).

Of the 2,311 markers that were originally assigned to the D subgenome of the CS reference genome, 1,517 were reassigned to the R subgenome of the Renan/Lo7 reference, 323 to the A subgenome, 304 to the B subgenome, and 167 were marked “Unassigned” (**Supplementary Table 4**). A total of 4,719 markers were originally missing a chromosomal location on the CS reference genome. Of these, 2,869 were reassigned to the R subgenome of the Renan/Lo7 reference, 138 to the A subgenome, 187 to the B subgenome, and 1,525 were marked as “Unassigned” (**Supplementary Table 4**).

### 3.3 Population structure analysis

**Figure 2:**
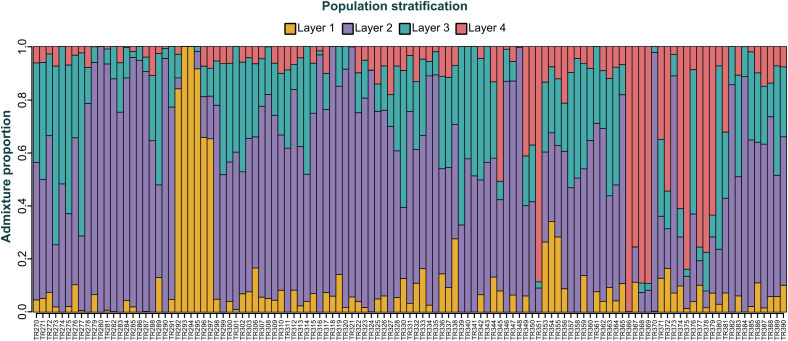
Cumulative admixture proportions of each triticale accession to the four expected population layers. Given one triticale accession, the admixture proportion indicates the proportion of its alleles originating from a certain population layer.

Population structure analysis was performed with a MCMC method in order to estimate admixture proportions of the triticale accessions to the expected population layers. The analysis indicated a high degree of admixture of the triticale collection (**Figure 2**).

Layers 1 and 4 were rather different from the other two layers. Layer 1 comprised four accessions, all from Romania. Layer 4 comprised eight accessions, all of North American origin. A total of 37 accessions were assigned to Layer 2. No accession could be exclusively assigned to Layer 3 (**Supplementary Table 5**). A total of 68 accessions was defined as admixed. Admixed accessions shared mostly alleles originating from Layers 2 and 3, whereas alleles originating from Layers 1 and 4 contributed little to the overall admixture of these accessions (**Figure 2**, **Table 2**).

**Table 2:** Maximum, minimum and average admixture proportions to each population layer (i.e., Layer 1, Layer 2, Layer 3, Layer 4) of the 117 triticale accessions.

Summary statistics	Layer 1	Layer 2	Layer 3	Layer 4
Genotypes Layer 1 (N = 4)				
Maximum	1.000	0.065	0.089	0.029
Minimum	0.841	0.000	0.000	0.000
Mean	0.939	0.027	0.027	0.007
Genotypes Layer 2 (N = 37)				
Maximum	0.142	1.000	0.228	0.141
Minimum	0.000	0.706	0.000	0.000
Mean	0.031	0.846	0.093	0.030
Genotypes Layer 4 (N = 8)				
Maximum	0.113	0.133	0.146	0.998
Minimum	0.000	0.001	0.000	0.755
Mean	0.031	0.082	0.039	0.849
Admixed genotypes (N = 68)				
Maximum	0.658	0.700	0.690	0.635
Minimum	0.000	0.149	0.069	0.000
Mean	0.090	0.463	0.329	0.118

Genotypes are grouped by their assignment to a population layer. Admixed genotypes form a separate group.

### 3.4 Genome-wide association study and identification of candidate genes

**Figure 3:**
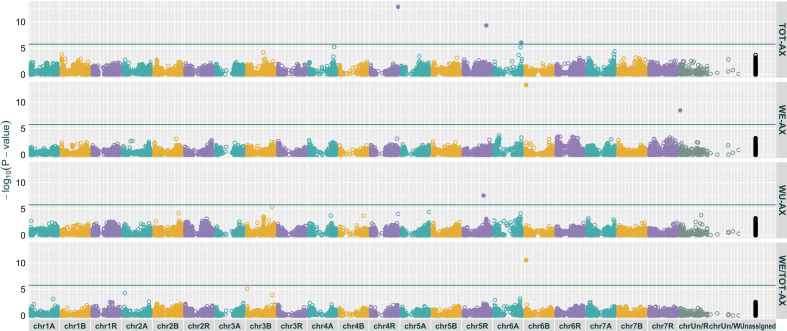
Manhattan plots showing the results of the GWAS for flour AX content of triticale. The abscissa represents the chromosomal coordinates of each tested SNP, whereas the ordinate represents the -*log*(*p*-value) per SNP as obtained from the BLINK model. The solid horizontal line represents the significance threshold α = 0.05 after Bonferroni correction. TOT-AX, total arabinoxylan content; WE-AX, water extractable arabinoxylan content; WU-AX, water-unextractable arabinoxylan content; WE/TOT-AX, proportion of WE-AX to TOT-AX.

**Figure 4:**
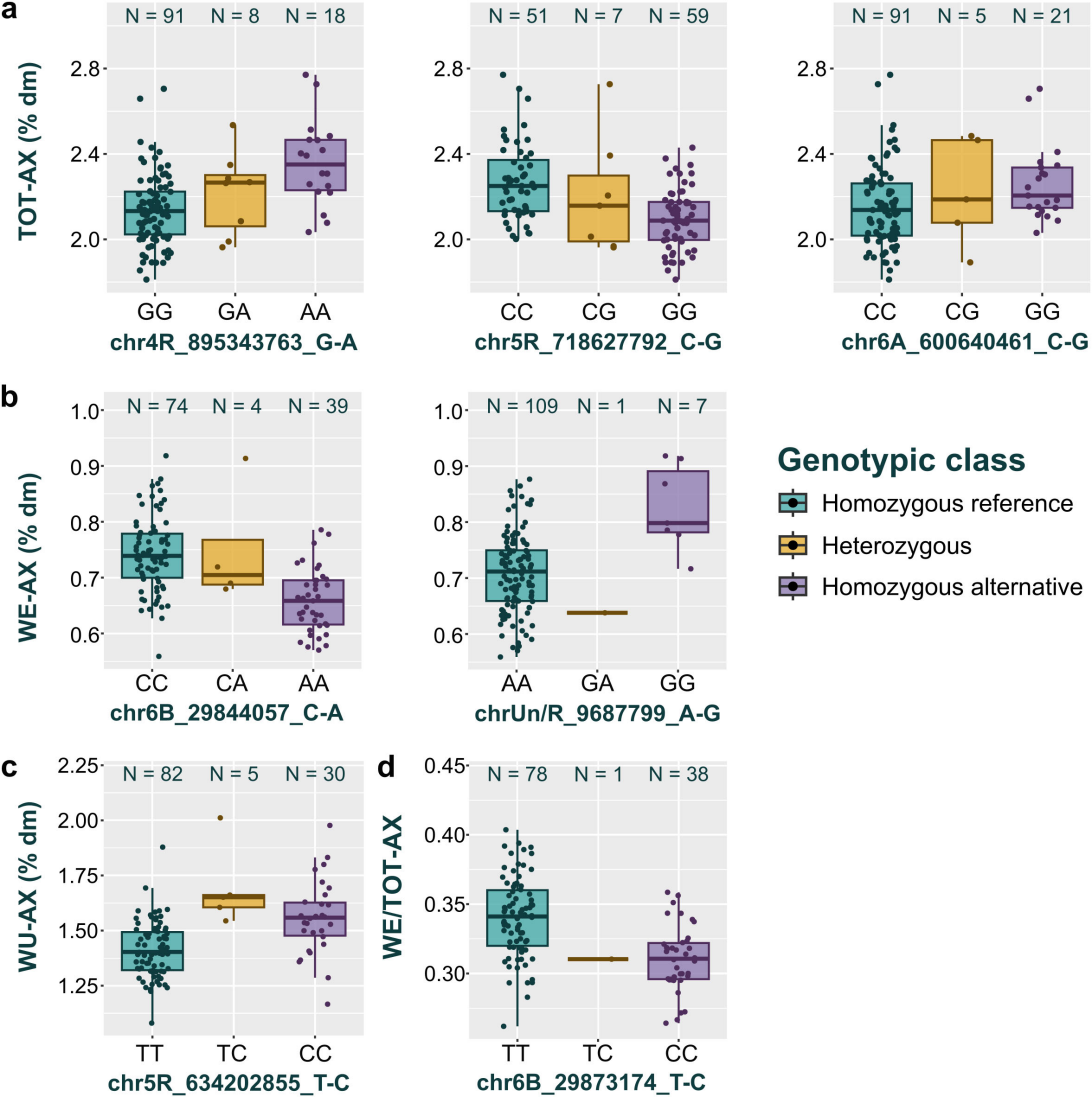
Boxplot distributions of total arabinoxylan content [TOT-AX; % of dry matter [dm], **(a)**], of water-extractable arabinoxylan content [WE-AX; %dm, **(b)**], of water-unextractable arabinoxylan content [WU-AX; %dm, **(c)**] and of the proportion of water-extractable arabinoxylan to total arabinoxylan [WE/TOT-AX; **(d)**] per associated SNP and stratified by genotypic class. The number of genotypes in each genotypic class is noted above the respective box.

The GWAS resulted in seven statistically significant MTAs (**Table 3**, **Figure 3**). Of these, three were associated with TOT-AX content, two with WE-AX content, one with WU-AX content, and one with WE/TOT-AX. The decreasing allele was the most frequent allele in the case of all MTAs for TOT-AX and WU-AX contents, and for the WE-AX MTA with SNP chrUn/R_9687799_A-G (**Figures 4a-c**). The increasing allele was the most frequent allele for the WE-AX MTA with SNP chr6B_29844057_C-A and for the WE/TOT-AX MTA with SNP chr6B_29873174_T-C (**Figures 4b, d**).

**Table 3:** List of statistically significant marker-trait associations (*p-*value < 0.05 after Bonferroni correction for multiple testing) for the four phenotypes tested.

Associated trait	SNP	Chromosome	Position (bp)	MAF	*p*-value	PVE (%)
TOT-AX	chr4R_895343763_G-A	4R	895,343,763	0.19	1.41×10^-13^	12.38
TOT-AX	chr5R_ 718627792_C-G	5R	718,627,792	0.47	4.82×10^-10^	7.08
TOT-AX	chr6A_600640461_C-G	6A	600,640,461	0.20	8.58×10^-7^	4.14
WE-AX	chr6B_29844057_C-A	6B	29,844,057	0.35	5.39×10^-14^	15.60
WE-AX	chrUn/R_9687799_A-G	Un^a^/R subgenome	9,687,799^b^	0.06	3.50×10^-9^	38.16
WU-AX	chr5R_634202855_T-C	5R	634,202,855	0.28	2.88×10^-8^	47.46
WE/TOT-AX	chr6B_29873174_T-C	6B	29,873,174	0.33	2.89×10^-11^	30.47

^a^Concatenated scaffolds that were not anchored to one of the seven pseudomolecules of the rye “Lo7” genome assembly.

^b^Positions on chrUn do not represent an actual physical position on the genome. SNPs are coded based on chromosome, position in base pairs (bp), and nucleotide change. TOT-AX, total arabinoxylan content; WE-AX, water-extractable arabinoxylan content; WU-AX, water-unextractable arabinoxylan content; WE/TOT-AX, proportion of WE-AX to TOT-AX; MAF, minor allele frequency; PVE, phenotypic variance explained.

Several genes related to grain filling were found within LD decay range of SNP chr4R_895343763_G-A (**Supplementary Tables 6**, **7**). These included three genes encoding *WALLS ARE THIN 1* (WAT1) related proteins, and four genes encoding SWEET sugar transporters (**Supplementary Table 7**).

Two unlinked MTAs were found on the long arm of chromosome 5R (**Table 3**; **Supplementary Table 6**), possibly defining two independent loci affecting TOT-AX and WU-AX. Among the genes annotated with a cell wall-related function within LD decay range of SNP chr5R_718627792_C-G there were 12 genes encoding uridine 5’-diphospho- (UDP) glycosyltransferases and one gene encoding a glucan endo-1,3-β-glucosidase (**Supplementary Tables 6**, **7**). Furthermore, four genes annotated as members of the Gibberellin-regulated protein 1 family (i.e., Snakin/GASA proteins; also known as GASR and GAST) were also found surrounding SNP chr5R_718627792_C-G. Four genes with cell wall-related functions, an UDP-glycosyltransferase, a peroxidase, a GH9 endonuclease, and a glucan endo-1,3-β-glucosidase, could be found within LD decay range of SNP chr5R_634202855_T-C (**Supplementary Tables 6**, **7**).

One gene encoding a hydroxycinnamoyl CoA quinate transferase and three genes encoding for Cinnamoyl CoA reductases were found within the LD decay range of SNP chr6A_600640461_C-G (**Supplementary Tables 6**, **7**).

The two MTAs for WE-AX and WE/TOT-AX colocalised on the short arm of chromosome 6B at a distance of 29,117 bp from each other (**Figure 3**, **Supplementary Tables 6**, **7**). Six genes found within LD decay range from either MTAs were annotated with cell wall-related functions (**Supplementary Tables 6**, **7**). Among these are the genes TraesRN6B0100087700 and TraesRN6B0100087800 corresponding with the wheat peroxidases PER1 and PER2.

One MTA for WE-AX with a major effect was detected on chrUn of the R subgenome (**Table 3**, **Figure 3**). Seven genes with cell wall-related functions were found within LD decay range of SNP_chrUn/R_A-G (**Supplementary Tables 6**, **7**). These genes are annotated with functions including UDP-glycosyltransferase, endo-1,4-beta-xylanse, glycosyltransferase, and hexosyltransferase.

## 4 Discussion

### 4.1 Arabinoxylan from the endosperm of triticale is characterised by less of the water-extractable fraction and more of the water-unextractable fraction as compared to wheat

Previous studies have shown triticale wholemeal to have higher AX contents than wheat wholemeal. It is interesting to make the same comparison between wheat and triticale flours. However, comparing the TOT-AX content in flours of triticale obtained in this study with those reported in wheat flour is not straightforward, since varying ranges have been reported depending on the composition of the wheat panel. Moreover, most authors report the range of phenotypic trait values, sometimes across multiple trials. For example, Gebruers et al. (2008) reported a TOT-AX content in wheat flour of 1.90% dm on average, and as high as 2.75% dm in a screen of 131 winter wheat cultivars during one season. Whereas Török et al. (2019), reported the range of TOT-AX content to be 1.03-3.90% dm in a screen of 41 winter wheat cultivars and breeding lines across three growing seasons. Using these values as reference, it appears that the TOT-AX content in the endosperm of triticale does not exceed that of wheat (**Table 1**).

Besides the TOT-AX content, it is also interesting to compare the contents of WE-AX and WU-AX fractions in triticale and wheat flours. In wheat white flour, Gebruers et al. (2008), reported WE-AX contents in the range of 0.30–1.40% dm. Whereas, Török et al. (2019), reported WE-AX contents in the range of 0.55-1.94% dm across three years of trials. Thus, compared to AX of wheat, the AX found in the endosperm of triticale is characterised by a lower proportion of the WE-AX fraction and, consequently, a higher proportion of the WU-AX fraction (**Table 1**). In the present study the content of FA was not investigated. Nevertheless, a determinant structural feature of WU-AX is the presence of diferulate bridges and a higher decoration by FA (Saulnier et al., 2007). Thus, the higher WU-AX content in the endosperm of triticale suggests a higher FA content in triticale flour as compared to wheat.

### 4.2 Using a custom ABR reference refines and increases the completeness of the projection of the DArTseq markers across the genome of triticale

In triticale, segregating populations have been used to determine the expected chromosomal location of the obtained molecular marker sequences (Losert et al., 2017a; Neuweiler et al., 2020, Neuweiler et al., 2021; Trini et al., 2021). However, in absence of such a segregating population, choosing the reference genome – or genomes – to position the obtained molecular markers remains non-trivial. Projecting marker sequences on an ABR reference such as the Renan/Lo7 reference used in the present study should allow to well represent the actual physical distribution of markers across the subgenomes of triticale. Although, introgression of single chromosomes of the wheat D subgenome in triticale are well documented and can involve any of the A, B, and R subgenomes (Ammar et al., 2004; Chen et al., 2019; Feng et al., 2019; Oettler, 2005; Sodkiewicz et al., 2011), European winter triticale is thought to generally be “complete” triticale (Tams et al., 2004). Thus, the incidence of D-substitutions can be expected to be negligible in the triticale collection used in the present study (**Supplementary Table 1**).

The observed increase in number of markers with a known chromosomal location was not only due to new projections on the R subgenome of the Renan/Lo7 reference, but also to projections on the A and B subgenomes (**Supplementary Table 4**). This is possibly explained by the fact that “Renan” is a French winter wheat variety, whereas “Chinese Spring” is a Chinese spring wheat variety. Thus, a higher genetic similarity can be expected between the winter triticale included in the collection used in the present study and the wheat variety “Renan”. However, there were still markers with a true missing BLASTn match (**Supplementary Table 4**). This suggests at the same time that the Renan/Lo7 reference still does not reflect the entire genetic constitution of triticale, and that polymorphisms independent of the wheat and rye ancestry may have arisen in the triticale gene pool in the process of allopolyploidisation (Ma and Gustafson, 2008).

### 4.3 The frequency of heterozygous calls should be harmonised with the expected reproductive behaviour of triticale

Despite the fact that triticale is generally assumed to be a self-pollinating species (Ammar et al., 2004; Oettler, 2005), when the DArTseq panel was filtered only by call-rate and MAF, several of the remaining markers showed a high degree of heterozygosity (**Supplementary Figure 1**). This is not just the case for the panel used in this study. In fact, a high frequency of heterozygous calls per marker can also be observed in DArTseq panels of other self-pollinating species such as wheat and common bean (*Phaseolus vulgaris* L.; Ates et al., 2018; Tyrka et al., 2021). The frequency of heterozygous calls per locus is rarely used as a filtering parameter in plant genomic studies using DArTseq, and no standard practice seems to have been established, with some authors opting for a hard threshold or for manually curating the genotype calls (Pascual et al., 2020; Sansaloni et al., 2020). Heterozygosity in self-pollinating species can arise from spontaneous cross-pollination. For example, in triticale, spontaneous cross-pollination has been reported to occur even at high rates depending on the growing conditions (Hughes et al., 1976; Sowa and Krysiak, 1996). However, averaged over loci, the individuals in the panel are predominantly homozygous, suggesting no recent spontaneous cross-pollination. Thus, the high frequency of heterozygous calls may represent “apparent” heterozygosity – an artifact arising from the genomic composition of triticale and the genotyping technique used.

There are several ways in which the genomic composition of triticale can bias the SNP calls: from copy number variation (Lloyd et al., 2018; Schiessl et al., 2019; Yaakov et al., 2013), to the relatedness among the subgenomes of triticale (Juery et al., 2021; Pont et al., 2013). Whereas copy number variation should be detectable by analysing read counts, inference of homoeologous sequences requires either wet-lab techniques such as probe hybridization or computational approaches such as comparative mapping (Glover et al., 2016; Wang et al., 2014). Given that the exact protocols of DArTseq are proprietary, it remains difficult to pin-point the exact source of the observed high heterozygosity of certain markers. Thus, in this work it is proposed that markers should be filtered based on their distribution of calls (i.e., either all genotypic classes are present, or only the two homozygous classes are present) and based on the proportion of heterozygous calls (i.e., proportion of heterozygous calls < proportion of either homozygous calls). Both criteria try to harmonise the distribution of calls per locus with the expected reproductive behaviour of triticale as well as with the constitution of the collection.

### 4.4 Admixture proportions reflect the breeding trends of triticale

The obtained admixture proportions reflect the breeding trends and practices of triticale. Breeding companies have established their triticale breeding pools starting from two relatively closed genetic pools: the CIMMYT triticale nursery and the Polish material (Ammar et al., 2004; Banaszak and Marciniak, 2002). This is thought to lead to a general admixture of the world-wide triticale germplasm (Losert et al., 2017b). Indeed, more than half of the triticale accessions was classified as admixed (**Supplementary Table 5**). At the same time, triticale is a young species from an evolutionary point of view, and its evolution is constrained within local breeding programmes. Such breeding programmes usually exploit local wheat and rye varieties in order to develop triticale varieties with optimal adaptation to the local environment (Ittu and Săulescu, 1996, Ittu and Săulescu, 2002). This can create quite isolated genetic groups, such as Layers 1 and 4 in the present study (**Figure 2**; **Table 2**).

### 4.5 The GWAS highlights larger effects of the rye genome on the arabinoxylan content in flour of triticale

The addition of chromosomes 4 or 5 of rye has already been shown to determine an increase in AX content in disomic addition lines of wheat (Boros et al., 2002; Szakács et al., 2016). In line with these results, the GWAS detected two MTAs for TOT-AX located on chromosomes 4R and 5R, respectively. These MTAs explained a larger proportion of the phenotypic variance of TOT-AX compared to the MTA for TOT-AX located on chromosome 6A (**Table 3**). Thus, it seems that the R subgenome of triticale is shifting the TOT-AX content in triticale flour towards the higher end of the range (**Figure 4a**).

Lovegrove et al. (2020), describe a wheat QTL for relative viscosity of flour aqueous extract – a proxy for WE-AX content – at a position comparable with that of the two MTAs for WE-AX and WE/TOT-AX that were found in the present study (**Table 3**, **Figure 3**). It is proposed that these two MTAs coincide with this wheat 6BS QTL. In fact, the genes TraesRN6B0100087700 and TraesRN6B0100087800, which correspond with the wheat peroxidases PER1 and PER2, are among the candidate genes within LD decay range of SNPs chr6B_29844057_C-A and chr6B_29873174_T-C. PER1 and PER2 have been described as the causal genes for the phenotype associated with the 6BS QTL of wheat (Mitchell et al., 2026). Several authors describe a major QTL for WE-AX located on the long arm of chromosome 1B of bread wheat (Ibba et al., 2021; Lee et al., 2023; Lovegrove et al., 2020; Martinant et al., 1999; Quraishi et al., 2011). However, no MTA for WE-AX was found in this region in the present study. It can be argued that this is due to the mostly European origin of the accessions in the triticale collection. In fact, the 1BL QTL appears to be a characteristic of Asian wheat material, whereas the 6BS QTL appears to be a characteristic of material with a European origin (Lee et al., 2023; Lovegrove et al., 2020).

Only one MTA for WE-AX, chrUn/R_9687799_A-G, was detected on the rye-derived subgenome of triticale. This MTA explains more than twice the phenotypic variance as explained by MTA chr6B_29844057_C-A. It is noteworthy that among the seven genotypes that are homozygous for the increasing allele of chrUn/R_9687799_A-G, there are five of the six Romanian accessions, which constitute a particularly distinct population layer (**Figure 2**, **Table 2**). Thus, this MTA detected on chrUn/R should be evaluated with caution as it may be an artifact of the high relatedness between these Romanian genotypes.

### 4.6 Arabinoxylan content in triticale flour and the balance between arabinoxylan fractions appear to be largely affected by the metabolism of hydroxycinnamic acids

The biosynthesis of AX does not seem to be a major driver of the variation of AX content in triticale. Only one of the candidate genes encoding a glycosyltransferase of family 61 (i.e., SECCEUnv1G0532750) has been found to be moderately expressed in the rye endosperm during grain development (Kozlova et al., 2022). Furthermore, none of the glycosyltransferases and glycoside hydrolases (GH) that are known to be involved in the synthesis and degradation of AX were among the candidate genes within LD range of the MTAs detected in this study. Instead, members of the GH9 and GH17 families were among such candidates. Most notably, gene SECCE5Rv1G0342380, which encodes a GH9 endoglucanase, has been shown to be expressed in the endosperm of rye, particularly at milk stage (Kozlova et al., 2022). However, the GH9 and GH17 families are not thought to be involved in the degradation of AX, although GH9 endoglucanases have also been previously detected in a GWAS for AX in tetraploid wheats (Marcotuli et al., 2015). GH9 endoglucanases have been implicated in the degradation of (1 → 3)(1 → 4)-β-D-glucan (Minic and Jouanin, 2006). These associations raise the question of whether there is a cross-talk between the biosynthesis and degradation of AX and (1 → 3)(1 → 4)-β-D-glucan.

Conversely, the biosynthesis of hydroxycinnamic acids and the cell wall decoration with such compounds appears to play a pivotal role in the determination of AX content and of its fractions in triticale flour. Peroxidases such as PER1 and PER2 affect the balance between WE-AX and WU-AX fractions in wheat by mediating the AX decoration with FA (Mitchell et al., 2026). Other enzymes that have been implicated in the feruloylation of AX include hydroxycinnamoyl CoA shikimate/quinate transferase and cinnamoyl CoA reductases (Chandrakanth et al., 2023; Freeman et al., 2017), which were abundant among the candidate genes identified in this study.

### 4.7 Future perspectives for arabinoxylan-targeted breeding in triticale

Future perspectives on AX-targeted breeding in triticale largely depend on the desired end use. In the context of animal husbandry, AX is generally considered as an antinutritional compound (Bautil et al., 2020; Petry et al., 2021). Whereas, in the context of human nutrition, a higher intake of DF compounds such as AX is strongly encouraged by health authorities (EFSA Panel on Dietetic Products, Nutrition, and Allergies (NDA), 2010). Moreover, AX, and particularly WE-AX, are reported to be positively correlated with bread making quality parameters of triticale flour (Chavoushi et al., 2022; Woś and Brzeziński, 2015). Thus two opposite breeding goals can be defined regarding AX content in triticale. The MTAs identified in this study can serve both. Notably, in this collection, the genotype homozygous for the decreasing allele was the most frequent genotype of the three MTAs for TOT-AX content (**Figure 4a**). Considering that triticale continues to be used mainly as feed, it can be speculated that the triticale genotypes comprising this collection have been under negative selection for AX content.*H²* estimates suggest that it is possible to invert this trend and steer the AX content in triticale flour via selection. In this collection, TOT-AX and WU-AX contents were more stable traits (*H²_TOT-AX_* = 0.82, *H²_WU-AX_* = 0.79) than WE-AX content and WE/TOT-AX (*H²_WE-AX_ =* 0.68, *H²_WE/TOT-AX_* = 0.60). Nevertheless, the availability of KASP markers for the wheat 6BS QTL for WE-AX content and WE/TOT-AX (Mitchell et al., 2026) makes WE-AX content and WE/TOT-AX more accessible breeding targets. The 6BS QTL was common in the collection (**Figures 4b, d**). However, three cultivars carrying this QTL were particularly outstanding in terms of their WE-AX content and WE/TOT-AX, namely “Fidelio”, “Stil”, and “Cultivo” (**Supplementary Tables 2**, **3**). These cultivars deserve further attention for the transfer of the wheat KASP markers in triticale. Targeting TOT-AX and WU-AX content in triticale still requires the validation of the detected MTAs and marker development. Considering their relatively large effects, SNPs chr4R_895343763_G-A and chr5R_634202855_T-C can be proposed as the most interesting candidates for marker development for TOT-AX and WU-AX content, respectively (**Table 3**). Four genotypes, namely “Trical Flex 719”, “Trical 135”, “Trical 158”, and “Trical 115”, are interesting as “increasing parents” for TOT-AX and WU-AX content, since they are carriers of the increasing alleles of both SNPs (**Supplementary Table 3**).

## 5 Conclusions

In the tested 118 triticale cultivars and breeding lines, the TOT-AX content in flour did not exceed the ranges reported for wheat flour. Nevertheless, the triticale flour was characterised by a higher WU-AX content as compared to wheat. This suggests a higher content of FA in triticale flour, which makes it interesting as a functional food ingredient. The GWAS of TOT-AX, WE-AX, and WU-AX contents, and WE/TOT-AX detected seven MTAs spread between the A, B, and R subgenomes of triticale. The SNPs on the R subgenome had larger effects on the associated traits compared to the SNPs on the A and B subgenomes. This makes these SNPs more amenable to the development of markers for the selection of AX-related traits in triticale. The presented results are relevant for the feed and food sector alike. In the context of breeding for food, the 6BS QTL for WE-AX content and WE/TOT-AX, SNPs chr4R_895343763_G-A and chr5R_634202855_T-C are of prime interest for the establishment of an AX-targeted breeding programme in triticale.

## Data Availability

The dataset used to perform the GWAS presented in this article can be found at the Zenodo online repository: 10.5281/zenodo.14906313.
