# Supplementary material for: A genome-wide association study of arabinoxylan content in flour of triticale (×Triticosecale Wittmack)
Source: Front Plant Sci. 2026 Apr 27;17:1809699. doi: 10.3389/fpls.2026.1809699 (PMC13158209; doi:10.3389/fpls.2026.1809699)

Supplementary Material


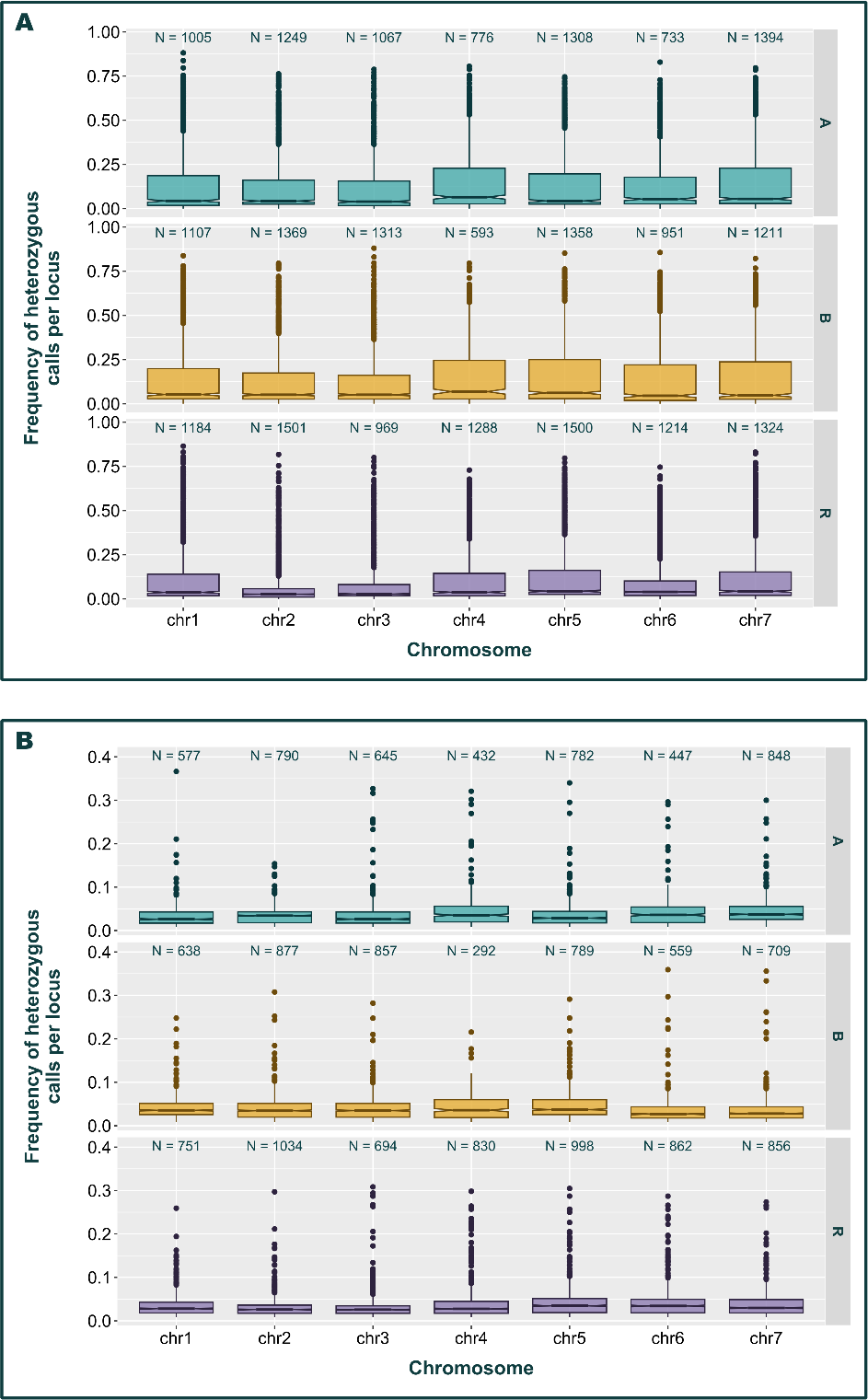
**Supplementary Figure 1.** Notched boxplots of the distribution of the frequency of heterozygous calls per locus stratified by subgenome (i.e., A, B, and R) and by chromosome group (chr1 to chr7: chromosome group 1 to chromosome group 7). The number of SNPs per chromosome group, per subgenome (N) is plotted on top of the corresponding boxplot. Frequency of heterozygous calls per locus of loci without a genomic projection is not shown. **a**, Subset of 29,633 markers obtained by filtering by call-rate and MAF only. **b**, Subset of 17,891 markers remaining after filtering by call-rate, MAF, and SNP heterozygosity.

**Supplementary Figure 2.** QQ-plot of the observed versus expected distribution of the -log(p-values) obtained from the GWAS for flour AX content of triticale. Different colours indicate the distribution of p-values obtained when testing against a differe

nt phenotype. TOT-AX: total arabinoxylan content (%, dry matter (dm)), WE-AX water-extractable arabinoxylan content, WU-AX: water-unextractable arabinoxylan content, WE/TOT-AX: proportion of WE-AX to TOT-AX.


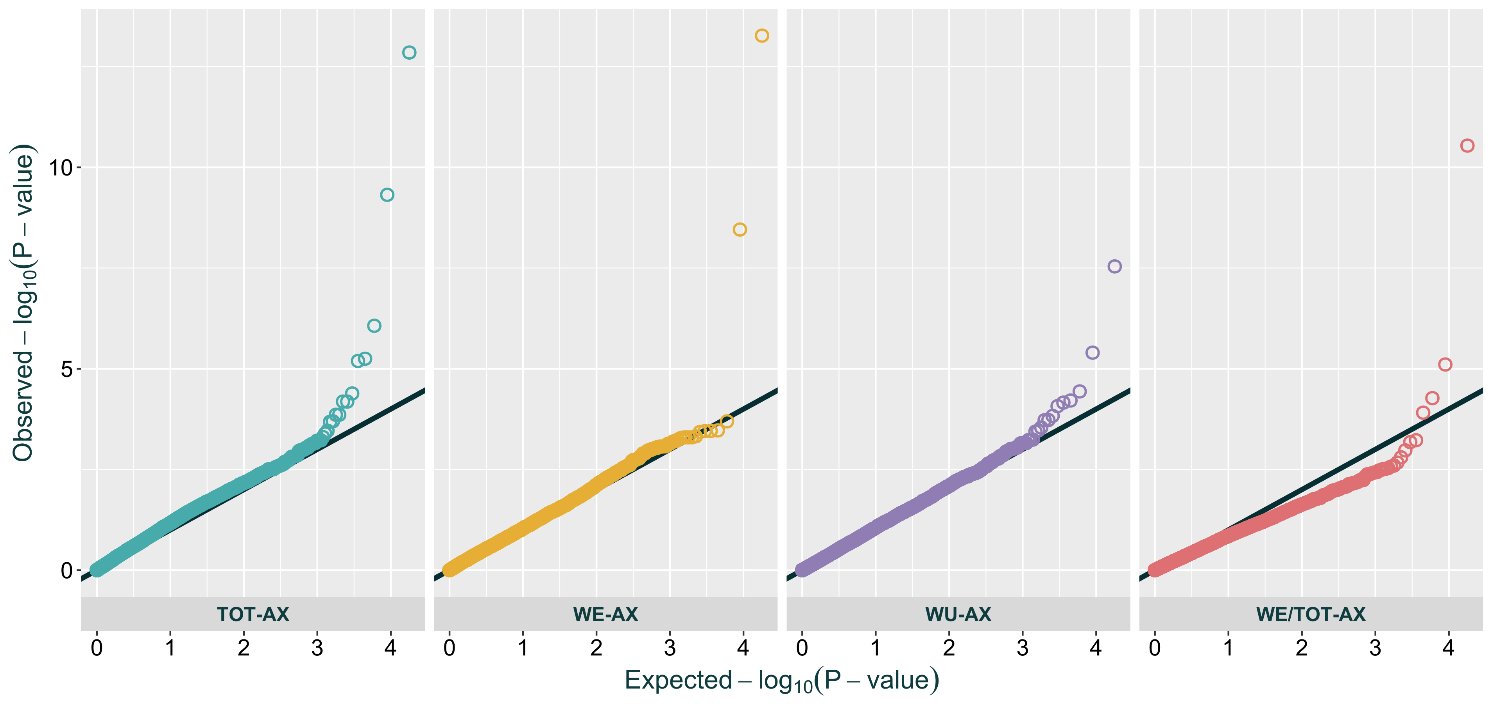

Supplement: Supplementary file 1 [file DataSheet1.docx]
